# Social Isolation/Loneliness and Tooth Loss in Community-Dwelling Older Adults: The Sukagawa Study

**DOI:** 10.1093/geroni/igad065

**Published:** 2023-06-26

**Authors:** Sei Takahashi, Toru Naganuma, Noriaki Kurita, Kenji Omae, Tsuyoshi Ohnishi, Takashi Yoshioka, Fumihito Ito, Taro Takeshima, Shingo Fukuma, Sugihiro Hamaguchi, Shunichi Fukuhara, Sukagawa Study Group, Takeshi Hasegawa, Nobuyuki Yajima, Kakuya Niihata, Hidekazu Iida, Susumu Kobayashi, Sho Sasaki, Hiroki Nishiwaki, Ryoji Tominaga

**Affiliations:** Department of General Internal Medicine, Fukushima Medical University, Fukushima, Japan; Futaba Emergency and General Medicine Support Center, Fukushima Medical University, Fukushima, Japan; Department of General Internal Medicine, Fukushima Medical University, Fukushima, Japan; Futaba Emergency and General Medicine Support Center, Fukushima Medical University, Fukushima, Japan; Department of Innovative Research and Education for Clinicians and Trainees (DiRECT), Fukushima Medical University Hospital, Fukushima, Japan; Department of Clinical Epidemiology, Graduate School of Medicine, Fukushima Medical University, Fukushima, Japan; Department of Clinical Epidemiology, Graduate School of Medicine, Fukushima Medical University, Fukushima, Japan; Center for Innovative Research for Communities and Clinical Excellence (CiRC2LE), Fukushima Medical University, Fukushima, Japan; Center for Innovative Research for Communities and Clinical Excellence (CiRC2LE), Fukushima Medical University, Fukushima, Japan; Department of Nephrology, Kasukabe Chuo General Hospital, Saitama, Japan; Center for Innovative Research for Communities and Clinical Excellence (CiRC2LE), Fukushima Medical University, Fukushima, Japan; Department of Preventive Medicine and Public Health, School of Medicine, Keio University, Tokyo, Japan; Department of Clinical Epidemiology, Graduate School of Medicine, Fukushima Medical University, Fukushima, Japan; Center for Innovative Research for Communities and Clinical Excellence (CiRC2LE), Fukushima Medical University, Fukushima, Japan; Center for Innovative Research for Communities and Clinical Excellence (CiRC2LE), Fukushima Medical University, Fukushima, Japan; Center for University-wide Education, School of Health and Social Services, Saitama Prefectural University, Saitama, Japan; Human Health Sciences, Graduate School of Medicine, Kyoto University, Kyoto, Japan; Department of General Internal Medicine, Fukushima Medical University, Fukushima, Japan; Center for Innovative Research for Communities and Clinical Excellence (CiRC2LE), Fukushima Medical University, Fukushima, Japan; Center for Innovative Research for Communities and Clinical Excellence (CiRC2LE), Fukushima Medical University, Fukushima, Japan; Department of Healthcare Epidemiology, School of Public Health in the Graduate School of Medicine, Kyoto University, Kyoto, Japan

**Keywords:** Frailty, Loneliness, Oral health, Psychosocial, Social support

## Abstract

**Background and Objectives:**

The relationship between social isolation/loneliness and oral health is unclear. This study investigated the association between social isolation/loneliness and tooth loss in older Japanese adults.

**Research Design and Methods:**

This was a cross-sectional study of a population-based cohort (the Sukagawa Study); 5,490 cohort study participants aged ≥75 years and who were independent answered a self-administered questionnaire in 2018. Social isolation was defined based on the 6-item Japanese version of the Lubben Social Network Scale. Loneliness was measured by the 3-item Japanese version of the University of California, Los Angeles (UCLA) Loneliness Scale version 3. The primary outcome was tooth loss, defined as having fewer than 20 teeth. The secondary outcomes were decreased toothbrushing frequency and diminished ability to chew food. Prevalence ratios (PRs) were estimated using a modified Poisson regression analysis in 2 models—Model 1, which adjusted for age, gender, smoking status, alcohol consumption, low annual income, and short education period, and Model 2, which added history of depression, history of diabetes mellitus, history of stroke, and cognitive impairment to Model 1.

**Results:**

The primary analysis included 5,490 participants. Adjusted PRs of social isolation and loneliness for tooth loss (Model 1) were 0.97 (95% confidence interval [CI] 0.93–1.01) and 1.07 (95% CI 1.02–1.12), respectively; those for decreased toothbrushing frequency were 1.17 (95% CI 0.98– 1.39) and 1.59 (95% CI 1.30–1.93), respectively; and those for chewing difficulty were 1.65 (95% CI 1.12–2.43) and 3.01 (95% CI 2.02–4.51), respectively. The adjusted PRs in Model 2 demonstrated results similar to that of Model 1.

**Discussion and Implications:**

Loneliness is associated with tooth loss among older adults, whereas social isolation is not. Our findings can inform plans for policymakers, professionals, and organizations to identify lonely older adults and provide social prescriptions to improve their access to oral health care services.

**Translational Significance:** Identifying the association between loneliness/social isolation and tooth loss can help prevent frailty in older adults and provide policymakers and caregivers with evidence-based guidelines for developing oral health interventions for this population. Our study demonstrated that loneliness in older adults is associated with tooth loss; however, social isolation was not found to be associated with tooth loss. Decreasing loneliness in an aging population may not only prevent tooth loss but also reduce negative health impacts.

## Background and Objectives

Social isolation and loneliness, which indicate a lack of social connection, are among the leading social determinants of health (Leigh-Hunt et al., 2017). People who are socially isolated or lonely are more likely to report poor mental and physical health (DiJulio et al., 2018), of which the impacts are comparable to other well-established health risk factors, such as smoking, alcohol, obesity, and low physical activity (Holt-Lunstad et al., 2017). Social isolation reflects an objective and quantitative lack of network size, whereas loneliness indicates a subjective and qualitative evaluation of the infrequency of contact with and scant closeness to others (Shankar et al., 2011). Previous studies have shown the association between social isolation and loneliness and salient geriatric outcomes, such as physical performance (Philip et al., 2020), depression (Nikmat et al., 2015), and mortality (Philip et al., 2020; Steptoe et al., 2013).

Oral health in older adults is represented by the absence of periodontal diseases and the number of remaining teeth. Its importance is highlighted by the association of poor oral hygiene with an increased risk of cardiovascular disease (Hansen et al., 2016), impaired physical function (Tada et al., 2003), and death (Koka & Gupta, 2018).

Theoretically, social isolation/loneliness can lead to poor dietary habits (Delerue Matos et al., 2021; Kobayashi & Steptoe, 2018; Locher et al., 2005; Umberson et al., 2010). High stress (Hawkley & Cacioppo, 2010; Umberson et al., 2010), failure of smoking cessation (Kobayashi & Steptoe, 2018), poor medical adherence (Holt-Lunstad & Smith, 2016), less frequent toothbrushing (Mathur et al., 2016), and less frequent dental visits (Mathur et al., 2016; Vozikaki et al., 2017), which can lead to poor oral health, which is one form of frailty. Recently, the association between social isolation or loneliness and oral health was also investigated (Koyama et al., 2021; Qi et al., 2023; Rouxel et al., 2017). Previous studies have found correlations between poor oral health-related quality of life and loneliness (Rouxel et al., 2017) and between oral health and social isolation (Koyama et al., 2021). A longitudinal study simultaneously examining the association between loneliness and social isolation and the number or loss of teeth in older adults found that only social isolation was associated with fewer teeth and tooth loss (Qi et al., 2023). Social isolation and loneliness are distinct constructs of social disconnection, which is supported by the fact that they are only moderately correlated (McHugh et al., 2017). In older individuals, either social isolation or loneliness may be present or may also overlap. Nonetheless, very few studies have examined social isolation and loneliness simultaneously (Koyama et al., 2021; Qi et al., 2023; Rouxel et al., 2017). Thus, a simultaneous assessment of both loneliness and social isolation would allow for a better understanding of the individual impact of these two factors on oral health (Cornwell & Waite, 2009; Holt-Lunstad et al., 2015) and would help inform comprehensive and effective policymaking. Moreover, research suggests that the quality of loneliness experience may vary across countries and cultures (Rokach et al., 2001). Therefore, exploring these matters specifically within the context of older Japanese adults with distinct and unique cultural backgrounds is essential.

Therefore, based on the hypothesis that both loneliness and social isolation lead to poor oral health, we analyzed the relationship between social isolation and loneliness, respectively, and oral health outcomes, using data from a large-scale population-based cohort with more than 6,000 older adults aged 75 years or older.

## Research Design and Methods

### Design and Setting

This cross-sectional study used data from the Sukagawa Study conducted in 2018. A detailed cohort profile of the Sukagawa Study has been reported in another study (Naganuma et al., 2021). The Sukagawa Study was a population-based cohort study that included community-dwelling, independent individuals aged 75 years or older (no upper age limit was set), and it was conducted to explore health-related quality of life, patient-reported outcomes, physical and psychological disabilities, and their associated factors among older adults in Fukushima, Japan. The study included functionally independent older adults who had been classified as long-term care insurance (LTCI) level 2 or lower and who had not been admitted to the hospital for longer than 6 months at the time of baseline. The LTCI is a mandatory social insurance system that provides long-term care to people with disabilities and is managed by the local municipalities in Japan. It grades people on seven levels according to the total estimated time required for their physical and mental care, from support level 1 (lowest) to care level 5 (highest; Tsutsui & Muramatsu, 2005). The study considered people with a care level of 2 or lower as mostly independent in their daily living activities. Participants would respond to annually issued self-administered questionnaires that included participants’ demographics, general health status, physical and mental functions, health-related behaviors, and socioeconomic status.

Cohort participants who had responded to the questionnaire in 2018 were included in the present study. All participants provided written informed consent. The study complied with the Declaration of Helsinki and was approved by the Research Ethics Committee of Fukushima Medical University School of Medicine (registered approval number: 2975).

### Oral Health Outcomes

This study’s primary outcome was self-reported tooth loss, defined as having 20 teeth or fewer. In the questionnaire, participants were asked, “How many teeth do you have left?” with four choices, “0,” “1–9,” “10–19,” and “20 or more.” We categorized the number of teeth as a clinically important dichotomous variable, “fewer than 20” and “20 or more,” as indicated by the relationship between annual medical expenditure and length of hospital stay (Naka et al., 2014; Shinsho, 2001).

The secondary outcomes were self-reported frequency of brushing teeth and a diminished ability to chew foods. To ascertain the frequency of brushing teeth and the ability to chew foods, we used a single-item questionnaire that we had developed. The frequency of brushing teeth was determined by asking, “How many times a day do you brush your teeth?” with five choices: “Three times a day,” “Once or twice a day,” “Occasionally,” “Almost never,” and “Never.” The answers were categorized as a dichotomous variable, “Every day” (including “Three times a day” and “Once or twice a day”) and “Not every day” (including “Occasionally,” “Almost never,” and “Never”), as recommended in a previous study by the American Dental Association (2022; Davies et al., 2003). The diminished ability to chew food was determined by asking the question, “How well can you chew your foods?” which had four choices: “Can chew even hard foods,” “Can chew soft foods well,” “Difficulty in chewing even soft foods,” and “Cannot chew at all.” The answers were categorized into a dichotomous variable, “Can chew” (including “Can chew even hard foods” and “Can chew soft foods well”) and “Cannot chew” (including “Difficulty in chewing even soft foods” and “Cannot chew at all”).

### Exposure

This study’s exposure included social isolation and loneliness. Social isolation was measured by the six-item Japanese version of the Lubben Social Network Scale (LSNS-6; Kurimoto et al., 2011). The construct validity of the LSNS-6 has been examined, and its Cronbach’s alpha coefficient has shown high internal consistency reliability of 0.82 (Kurimoto et al., 2011). Its total score ranged from 0 to 30, and a score of <12 was defined as socially isolated according to the original study (Lubben et al., 2006). Loneliness was measured using the three-item Japanese version of the University of California, Los Angeles (UCLA) Loneliness Scale version 3 (Igarashi, 2019). The scale’s unidimensionality has been validated, and it has shown high internal consistency reliability with a Cronbach’s alpha coefficient of 0.81 (Igarashi, 2019). Its score ranged from 3 to 9, with a score ≥6 defined as being lonely, according to a previous study (Hughes et al., 2004).

### Covariates

**Figure 1.**
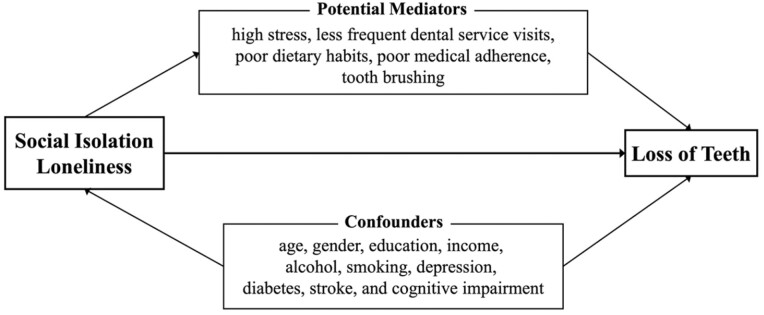
Conceptual model. The conceptual model depicts the hypothetical relationship among loneliness, social isolation, and tooth loss.

We considered the following factors as potential confounders: sociodemographic characteristics, such as age, gender (male or female), smoking status (current smoker or nonsmoker), alcohol consumption (≥3 or <3 days/week), annual income (<3,000,000 or ≥3,000,000 JPY/year [about 27,000 USD]), educational history (junior high school graduate and below or high school graduate and above), and comorbidities such as a history of depression, diabetes, stroke, or cognitive impairment. Cognitive impairment was assessed using the Mini-Cog test, which was included in the health survey in 2018; those with a score ≤2/5 were considered to have cognitive impairment (Borson et al., 2003). The aforementioned covariates were included as potential confounders as they had been adjusted for in previous studies (Koyama et al., 2021; Qi et al., 2023; Rouxel et al., 2017). The conceptual model (Figure 1) illustrates the relationship between the exposures, outcome, and covariates based on the directed acyclic graph (Supplementary Figure S1).

### Statistical Analysis

Regarding the participants’ characteristics, age is presented in mean and standard deviations and categorical variables are presented in numbers and percentages. We tested the association between loneliness and social isolation using Pearson’s chi-square test. The internal consistency reliability of the LSNS-6 and UCLA Loneliness scales was assessed using Cronbach’s alpha. To adjust for the potential confounders, modified Poisson regression models (Zou, 2004) were fit to estimate the prevalence ratios (PRs) and 95% confidence intervals (CIs) of the oral health outcomes. The model was chosen to directly estimate the PR of the primary outcome variable with a nonrare proportion. The aforementioned comorbidities can be considered as the cause or result of loneliness and social isolation and thus can play a role as confounding or intermediary factors in multivariate analyses. Therefore, we fitted two models—Model 1, which adjusted for sociodemographic factors only, and Model 2, which added comorbidities to Model 1. To ensure the accuracy of the results of the analysis, which adjusted for variables with a high percentage of missing data, we used the multiple imputation by chained equation method to complement the missing data of the exposures, outcomes, and covariates by 100 imputations, using predictive mean matching for continuous variables (LSNS-6, UCLA Loneliness Scale Short, and Mini-Cog). We ordered logistic regression for categorical variables (number of teeth, frequency of brushing teeth, ability to chew foods, smoking status, alcohol consumption, educational history, and annual income; Graham et al., 2007; UCLA: Statistical Consulting Group, 2022). We tested multiplicative and additive interactions between social isolation and loneliness, using their multiplicative term and the relative excess risk due to interaction (RERI), respectively (Knol & VanderWeele, 2012; Rothman et al., 2008). We conducted all analyses using the complete case for sensitivity analysis. All analyses were performed with Stata 16 (StataCorp. 2019, Stata Statistical Software: Release 16. College Station, TX: StataCorp LLC).

## Results

**Figure 2.**
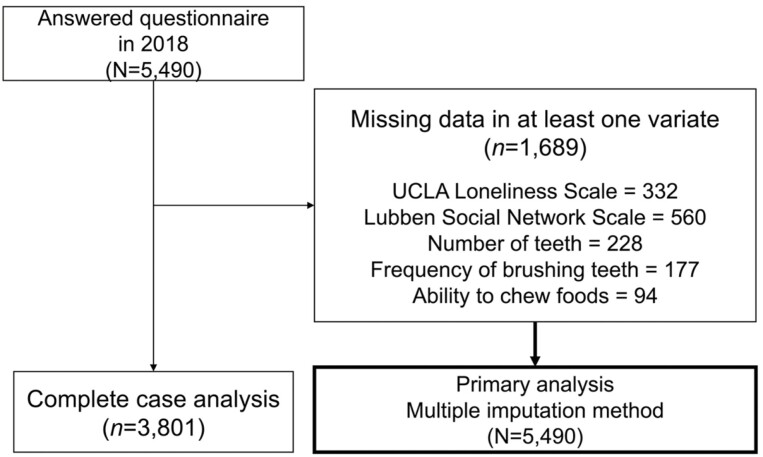
Study flow chart. UCLA = University of California, Los Angeles.

The study flow for this research is depicted in Figure 2. Questionnaires were sent to 8,869 eligible individuals aged 75 years and older in Sukagawa City in March 2018. Of the 5,490 participants who responded (response rate: 61.9%), all participants were included in the analysis.

The characteristics of the participants are summarized in Table 1. Among the 5,490 participants, 1,248 (25.3%) were socially isolated, and 595 (11.5%) were lonely; those who were socially isolated were more likely to feel lonely compared to those who were not (*p* <.001). Cronbach’s alpha coefficients were 0.88 for the LSNS-6 Scale and 0.77 for the UCLA Loneliness Scale. Of the participants, 3,785 (71.9%) had fewer than 20 teeth, 638 (12.0%) did not brush their teeth every day, and 148 (2.7%) could not chew food. Their mean age was 80.9 years, and the proportion of female participants was 57.9%. The education level for approximately half of the participants was junior high school and below. The proportion of female participants was lower among the socially isolated group than in the other groups. Compared to the other groups, socially isolated and lonely groups had a low income, a history of depression, and were current smokers. Compared to the participants who did not answer our questions related to social isolation and loneliness, the participants’ mean age was higher, and they had a higher percentage of missing measurements.

**Table 1.** Characteristics of Study Participants

Variable	Total (*N* = 5,490)	By social isolation			By loneliness		
		Not socially isolated (*n* = 3,682)	Socially isolated (*n* = 1,248)	Missing (*n* = 560)	Not lonely (*n* = 4,563)	Lonely (*n* = 595)	Missing (*n* = 332)
UCLA Loneliness Scale							
Not lonely	4,563 (88.5%)	3,350 (93.2%)	895 (74.6%)	318			
Lonely	595 (11.5%)	245 (6.8%)	305 (25.4%)	45			
Luben Social Network Scale							
Not socially isolated	3,682 (74.7%)				3,350 (78.9%)	245 (44.5%)	87
Socially isolated	1,248 (25.3%)				895 (21.1%)	305 (55.5%)	48
Age, mean (*SD*)	80.9 (4.9)	80.5 (4.7)	81.5 (5.4)	82.1 (5.0)	80.7 (4.8)	81.4 (5.1)	82.4 (5.1)
Gender							
Male	2,310 (42.1%)	1,506 (40.9%)	632 (50.6%)	172	1,937 (42.5%)	279 (46.9%)	94
Female	3,180 (57.9%)	2,176 (59.1%)	616 (49.4%)	388	2,626 (57.5%)	316 (53.1%)	238
Education period							
Junior high school graduate and below	2,782 (54.4%)	1,883 (53.4%)	639 (53.4%)	260	2,300 (52.7%)	346 (60.9%)	136
Income							
Less than 3,000,000 Yen/year	3,405 (72.8%)	2,293 (71.1%)	849 (76.4%)	263	2,871 (71.5%)	410 (79.6%)	124
History of diabetes mellitus	761 (13.9%)	466 (12.7%)	216 (17.3%)	79	596 (13.1%)	121 (20.3%)	44
History of stroke	166 (3.0%)	92 (2.5%)	57 (4.6%)	17	113 (2.5%)	33 (5.5%)	20
History of depression	109 (2.0%)	54 (1.5%)	45 (3.6%)	10	79 (1.7%)	24 (4.0%)	6
Mini-Cog test							
2 points or less	46 (6.2%)	29 (5.7%)	9 (5.1%)	8	36 (5.6%)	7 (8.5%)	3
Smoking status							
Current smoker	344 (6.6%)	229 (6.5%)	93 (7.7%)	22	286 (6.6%)	44 (7.7%)	14
High alcohol consumption							
3 days or more/week	1,117 (21.4%)	759 (21.6%)	266 (22.1%)	92	953 (21.8%)	117 (20.6%)	47
The number of teeth							
20 or more	1,477 (28.1%)	1,016 (28.6%)	338 (28.1%)	123	1,290 (29.2%)	127 (22.2%)	60
Less than 20	3,785 (71.9%)	2,540 (71.4%)	866 (71.9%)	379	3,125 (70.8%)	444 (77.8%)	216
Frequency of brushing teeth							
Not everyday	638 (12.0%)	387 (10.8%)	188 (15.5%)	63	478 (10.8%)	116 (20.2%)	44
Ability to chew foods							
Can’t chew	148 (2.7%)	75 (2.1%)	58 (4.7%)	15	86 (1.9%)	44 (7.5%)	18

*Notes*: *SD* = standard deviation; UCLA = University of California, Los Angeles. All percentages exclude missing responses. Among the 5,490 participants in this study, the age range was from 75 to 102 years old.

Table 2 presents the relationships between social isolation, loneliness, and tooth loss. Adjusted PRs of social isolation and loneliness for tooth loss were 0.97 (95% CI 0.93–1.01) and 1.07 (95% CI 1.02–1.12), respectively, in Model 1, and 0.97 (95% CI 0.93–1.01) and 1.06 (95% CI 1.01–1.11), respectively, in Model 2. We found insufficient evidence of an interaction between social isolation and loneliness on the additive and multiplicative scales (RERI 0.07 [95% CI −0.03 to 0.17] and ratio of PRs 1.07 [95% CI 0.97–1.18]). A similar trend was observed in the complete case analysis (CCA; Supplementary Table S1).

**Table 2.** Association Between Social Isolation, Loneliness, and Number of Teeth

Variable	Model 1^a^		Model 2^b^	
	PRs	95% CI	PRs	95% CI
Not socially isolated	1.0	Reference	1.0	Reference
Socially isolated	0.97	0.93–1.01	0.97	0.93–1.01
Not lonely	1.0	Reference	1.0	Reference
Lonely	1.07	1.02–1.12	1.06	1.01–1.11

*Notes*: CI = confidence interval; PR = prevalence ratio.

^a^Model 1: PRs are estimated via a modified Poisson regression model with adjustment of age, gender, smoking, alcohol consumption, education, and house income.

^b^Model 2: Model 1 + history of depression + history of diabetes + history of stroke + cognitive impairment.

Supplementary Table S2 shows the correlations between social isolation, loneliness, and infrequent toothbrushing. Adjusted PRs for social isolation and loneliness were 1.17 (95% CI 0.98–1.39) and 1.59 (95% CI 1.30–1.93), respectively, in Model 1, and 1.13 (95% CI 0.95–1.35) and 1.50 (95% CI 1.23–1.83), respectively, in Model 2. A similar trend was observed in CCA (Supplementary Table S3).

Supplementary Table S4 presents the associations between social isolation, loneliness, and having difficulty in chewing food. Both social isolation and loneliness were significantly associated with the declined ability to chew foods. Adjusted PRs were 1.65 (95% CI 1.12–2.43) and 3.01 (95% CI 2.02–4.51), respectively, in Model 1, and 1.63 (95% CI 1.10–2.41) and 2.94 (95% CI 1.96–4.43), respectively, in Model 2. The CCA showed no significant association between social isolation and chewing strength but showed a significant association with loneliness (social isolation: PR 1.31, 95% CI 0.81–2.12; loneliness: PR 2.59, 95% CI 1.55–4.32; Supplementary Table S5).

## Discussion and Implications

This cross-sectional study of people aged 75 years or older in Japan examined the correlation between loneliness or social isolation and tooth loss. We found that loneliness was significantly associated with fewer teeth, while social isolation was not. Further, unlike social isolation, loneliness was significantly associated with less frequent brushing of teeth. Both social isolation and loneliness were significantly associated with the reduced ability to chew foods. These results were consistent irrespective of whether the comorbidities, which could be both mediating and confounding factors, were included in the analysis. Our findings suggest that the lack of social connection, especially feeling lonely, has a negative impact on oral health.

The possible link between loneliness and tooth loss may be explained by the lack of oral self-care, such as toothbrushing, due to reduced physical activity. Some previous studies have shown that loneliness is associated with reduced physical activity, resulting in reduced capacity for the self-regulation of lifestyle behaviors (Hawkley & Cacioppo, 2010). Additionally, some studies have reported that loneliness may decrease health-related behaviors due to a lack of self-monitoring (Burr & Lee, 2013). There are some differences between our study’s results and those of previous studies. Although one study reported an association between oral health-related quality of life and loneliness, the oral health status was not directly measured by the number of teeth, and social isolation was not included in the model (Rouxel et al., 2017). Another cross-sectional study found that the number of remaining teeth was associated with social isolation (Koyama et al., 2021). Qi et al. (2023) reported on the association between social isolation, but not loneliness, and fewer remaining teeth and tooth loss, which was not observed in our study. These discrepancies might be attributed to the differences in social isolation measures. Our study used validated measures of loneliness and social isolation and included populations who were more vulnerable to losing teeth. Qi et al. (2023) measured loneliness using a single-item question, and their social isolation construct included life functions such as lack of social participation and help; therefore, the social isolation variable may have strongly influenced the participants’ health behaviors. Additionally, the narrow range of the loneliness scale used in this study may have resulted in misclassification of people feeling lonely as not being lonely, thus underestimating the association between loneliness and outcomes. However, as a significant association between loneliness and loss of teeth was demonstrated, we do not believe the impact of the misclassification bias is large. The disparities observed in the findings between our study and Qi’s study may also be attributed to the differential influence of social context on the experience of loneliness (Rokach et al., 2001). Chinese culture, with its extensive family networks and adherence to traditional Confucian-based filial piety (Sun et al., 2022), likely contributes to mitigating feelings of loneliness and poor oral health outcomes among older adults. In contrast, Japanese culture is characterized by a perception of social rigidity, which pertains to the perceived challenges of voluntarily joining or leaving social groups and establishing new social connections (Badman et al., 2022). This distinctive cultural background may hinder older adults from alleviating their feelings of loneliness and addressing their oral health issues. Our finding in the present study that some individuals are socially isolated but not lonely and others are not socially isolated but lonely supports that loneliness and social isolation are distinct constructs. Simultaneous examination of the association between the two and oral health will help us understand which of the two should be addressed in interventions to protect oral health.

Our study provides a rationale for policymakers and caregivers regarding their plans and actions for oral health intervention among lonely older adult populations. The concise questionnaire-based survey, in collaboration with the local government, allowed us to survey the entire community and identify socially disconnected older adults in a resource-limited setting. Our findings propose plans for policymakers, professionals, and organizations involved in oral health to identify lonely older adults and to provide social prescriptions to ease their access to oral health care services or provide targeted close contacts such as caregiving through at-home visits. This proposal should be formulated based on the cultural background of each country. Japan is facing changes in family structure, such as the shift to nuclear families and an increase in single-person households due to declining birth rates and the aging population (Cabinet Office, Government of Japan, 2021). This resulted in a 2.7-fold increase in the number of older single-person households between 1980 and 2019 (Cabinet Office, Government of Japan, 2021), which makes the Japanese more vulnerable to social isolation. Additionally, due to changes in local communities, such as a decrease in interaction and mutual support among community residents (Cabinet Office, Government of Japan, 2021), older people in Japan are living in a society prone to loneliness. This is also supported by an international survey conducted by the Cabinet Office, Japan, showing that older people in Japan have the fewest number of people they can rely on other than their living family members compared to other countries (Director General for Policy Coordination, Cabinet Office, 2020).

This study has several strengths. First, it examined the relationship between social isolation and tooth loss, and loneliness and tooth loss among older adults—a subject that has rarely been investigated in previous studies. Additionally, the results have high generalizability because this study used data from a population-based cohort with a large sample size and a high response rate (Naganuma et al., 2021). Furthermore, this study’s participants were subject to high risks of loneliness, isolation, and vulnerability to poor oral health because of their advanced age, which allowed us to examine them in a population with a sufficient proportion of exposure.

This study has several limitations. First, because it was a cross-sectional study and the pathway between loneliness/social isolation and poor oral health can be bidirectional (Abbas et al., 2023), the temporal relationship remains uncertain. For example, poor oral health, such as loss of teeth and reduced chewing ability, may discourage face-to-face conversation and eating, thereby inhibiting opportunities for social interaction with others. Additionally, diseases caused by poor oral health (e.g., depression and diabetes) may be mediating factors and cause loneliness and social isolation. Therefore, a population-based longitudinal study to assess this association with more specific oral health factors, such as dental caries and periodontal disease, beyond the number of teeth is required to verify our results. Second, the oral health status, measured as an outcome in this study, was obtained from the self-reported questionnaires only; thus, it may not reflect the actual number of teeth and other oral conditions. However, previous studies have shown that the actual status for self-reported oral health questions was valid (Matsui et al., 2016), and the consistency in the results of tooth loss and secondary outcomes suggests robust reliability of the observed association. Third, this study may have selection bias associated with nonparticipation because individuals who are socially isolated or lonely may be less likely to respond to the survey. The excluded group may have had lower health status and awareness at the time of response, thereby leading to a potential bias that resulted in underestimating the relationships. Additionally, as indicated in the pilot study showing that the proportion of isolation among those who did not respond to the self-administered LSNS-6 questionnaire was higher than that among those who did respond to it (Taylor et al., 2016), it is possible that individuals who did not respond to the questionnaire may be more likely to be socially isolated or lonely in the present study. However, because the response rate was relatively high (Naganuma et al., 2021), the effect of this bias on representativeness is expected to be minimal.

## Conclusion and Implications

We found that loneliness, rather than social isolation, was associated with tooth loss. The implication for future research is that further longitudinal research on the impact of loneliness and social isolation on oral health is required, which can help inform future health policy decisions by national and local governments.

## Supplementary Material

igad065_suppl_Supplementary_Material

## Data Availability

The data utilized in this article were provided by Sukagawa City with permission. The data used in this article will be shared upon reasonable request to the corresponding author with permission from Sukagawa City.
