# Correction to: Social Isolation/Loneliness and Tooth Loss in Community-Dwelling Older Adults: The Sukagawa Study

**DOI:** 10.1093/geroni/igad122

**Published:** 2023-11-07

This is a correction to: Sei Takahashi, Toru Naganuma, Noriaki Kurita, Kenji Omae, Tsuyoshi Ohnishi, Takashi Yoshioka, Fumihito Ito, Taro Takeshima, Shingo Fukuma, Sugihiro Hamaguchi, Shunichi Fukuhara and the Sukagawa Study Group, Social Isolation/Loneliness and Tooth Loss in Community-Dwelling Older Adults: The Sukagawa Study, *Innovation in Aging*, Volume 7, Issue 6, 2023, Page 1-8, https://doi.org/10.1093/geroni/igad065

In the originally published version of this manuscript, there were minor errors in the abstract, the main text, tables, and figure legends.

## Correction List

#1 Abstract
#2 Main text
#3 Table 2
#4 Supplementary Table S4
#5 Legend of Figure 1
#6 Legend of Figure 2

## #1 Abstract

p. 1 Abstract line 12: “**Results:** The primary analysis included 4,645 participants. Adjusted PRs of social isolation and loneliness for tooth loss (Model 1) were 0.97 (95% confidence interval [CI] 0.92–1.01) and 1.06 (95% CI 1.01–1.12), respectively; those for decreased toothbrushing frequency were 1.13 (95% CI 0.95–1.36) and 1.56 (95% CI 1.26–1.92), respectively; and those for chewing difficulty were 1.61 (95% CI 1.06–2.43) and 2.94 (95% CI 1.91–4.53), respectively.”

Corrected to: “**Results:** The primary analysis included 5,490 participants. Adjusted PRs of social isolation and loneliness for tooth loss (Model 1) were 0.97 (95% confidence interval [CI] 0.93–1.01) and 1.07 (95% CI 1.02–1.12), respectively; those for decreased toothbrushing frequency were 1.17 (95% CI 0.98– 1.39) and 1.59 (95% CI 1.30–1.93), respectively; and those for chewing difficulty were 1.65 (95% CI 1.12–2.43) and 3.01 (95% CI 2.02–4.51), respectively.”

## #2 Main text

p.3 column 2 line 48: “Among the 5,490 participants, 1,248 (25.0%) were socially isolated, and 595 (11.5%) were lonely; those who were socially isolated were more likely to feel lonely com- pared to those who were not (p <.001).”

Corrected to: “Among the 5,490 participants, 1,248 (25.3%) were socially isolated, and 595 (11.5%) were lonely; those who were socially isolated were more likely to feel lonely compared to those who were not (p <.001).”

## #3 Table 2

Table 2: Social isolated PR and 95% confidence intervals in Model 1 were 0.97 (0.92-1.01).

**Table 2.** Association Between Social Isolation, Loneliness, and Number of Teeth

	Model 1		Model 2	
	PRs	95% CI	PRs	95% CI
Not socially isolated	1.0	reference	1.0	reference
Socially isolated	0.97	0.93 – 1.01	0.97	0.93 – 1.01
Not lonely	1.0	reference	1.0	reference
**Lonely**	**1.07**	**1.02 – 1.12**	**1.06**	**1.01 – 1.11**

*Note*. CI: confidence interval, PR: prevalence ratio.

Model 1: PRs are estimated via a modified Poisson regression model with adjustment of age, gender, smoking, alcohol consumption, education, and house income.

Model 2: Model 1 + history of depression + history of diabetes + history of stroke + cognitive impairment.

Corrected to: Social isolated PR and 95% confidence intervals in Model 1 were 0.97 (0.93-1.01).

**Table 2.** *Association Between Social Isolation, Loneliness, and Number of Teeth*

**Table:** 

	Model 1		Model 2	
	PRs	95% CI	PRs	95% CI
Not socially isolated	1.0	reference	1.0	reference
Socially isolated	0.97	0.92 – 1.01	0.97	0.93 – 1.01
Not lonely	1.0	reference	1.0	reference
**Lonely**	**1.07**	**1.02 – 1.12**	**1.06**	**1.01 – 1.11**

*Note*. CI: confidence interval, PR: prevalence ratio.

Model 1: PRs are estimated via a modified Poisson regression model with adjustment of age, gender, smoking, alcohol consumption, education, and house income.

Model 2: Model 1 + history of depression + history of diabetes + history of stroke + cognitive impairment.

Corrected to:

## #4 Supplementary Table S4

Supplementary Table S4: Social isolated PR and 95% confidence intervals in Model 1 were 1.61 (1.12-2.43).

Corrected to: Social isolated PR and 95% confidence intervals in Model 1 were 1.65 (1.12-2.43).

**Snupplementary Table S4.** *Association Between Social Isolation, Loneliness, and Difficulty in Chewing*

**Table:** 

Variable	Model 1^a^		Model 2^b^	
	PRs	95% CI	PRs	95% CI
Not socially isolated	1.0	reference	1.0	reference
Socially isolated	**1.61**	**1.12 – 2.43**	**1.63**	**1.10 – 2.41**
Not lonely	1.0	reference	1.0	reference
Lonely	**3.01**	**2.02 – 4.51**	**2.94**	**1.96 – 4.43**

Note. PR = prevalence ratio, CI = confidence interval.

a Model 1: PRs are estimated via a modified Poisson regression model with adjustment of age, gender, smoking, alcohol consumption, education, and house income.

b Model 2: Model 1 + history of depression + history of diabetes + history of stroke + cognitive impairment.

Corrected to:

**Supplementary Table S4.** *Association Between Social Isolation, Loneliness, and Difficulty in Chewing*

**Table:** 

Variable	Model 1^a^		Model 2^b^	
	PRs	95% CI	PRs	95% CI
Not socially isolated	1.0	reference	1.0	reference
Socially isolated	**1.65**	**1.12 – 2.43**	**1.63**	**1.10 – 2.41**
Not lonely	1.0	reference	1.0	reference
Lonely	**3.01**	**2.02 – 4.51**	**2.94**	**1.96 – 4.43**

Note. PR = prevalence ratio, CI = confidence interval.

a Model 1: PRs are estimated via a modified Poisson regression model with adjustment of age, gender, smoking, alcohol consumption, education, and house income.

b Model 2: Model 1 + history of depression + history of diabetes + history of stroke + cognitive impairment.

## #5 Legend of Figure 1

Legend of Figure 1: “Figure 1. Conceptual model. The conceptual model depicts the hypothetical relationship among loneliness, social isolation, and tooth loss. UCLA = University of California, Los Angeles.”

Corrected to: “Figure 1. Conceptual model. The conceptual model depicts the hypothetical relationship among loneliness, social isolation, and tooth loss.”

## #6 Legend of Figure 2

Legend of Figure 2: “Figure 2. Study flow chart.”

Corrected to: “Figure 2. Study flow chart. UCLA = University of California, Los Angeles.”

The authors apologize for the errors.

