# Supplementary material for: Social Isolation/Loneliness and Tooth Loss in Community-Dwelling Older Adults: The Sukagawa Study
Source: Innov Aging. 2023 Jun 26;7(6):igad065. doi: 10.1093/geroni/igad065 (PMC10368321; doi:10.1093/geroni/igad065)
Supplement: igad065_suppl_Supplementary_Material [file igad065_suppl_supplementary_material.docx]

**Online Supplementary Material**


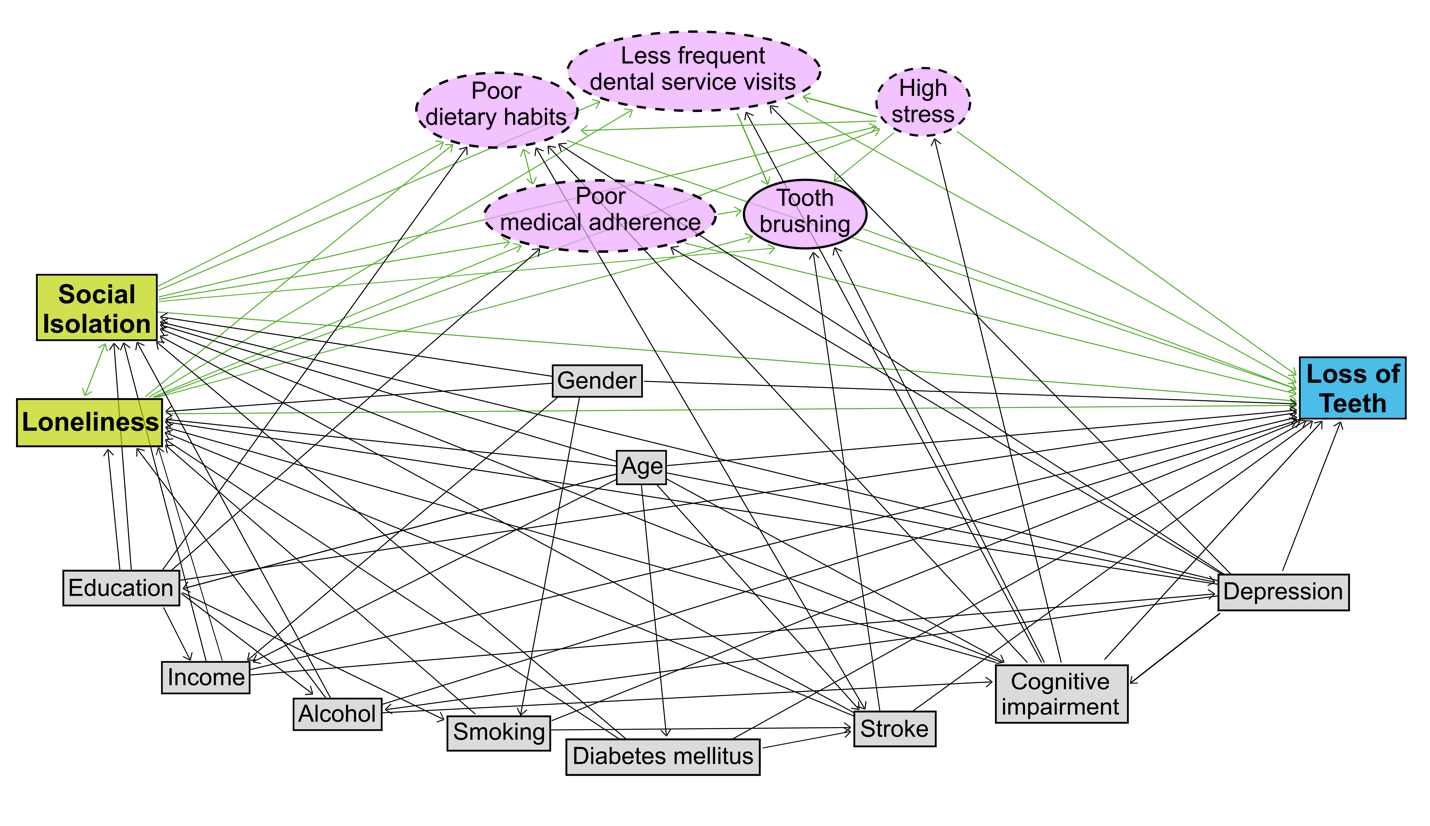


**Supplemental Figure S1.** Directed acyclic graph (DAG).

Notes. The DAG illustrates the hypothetical relationships among loneliness and social isolation as exposure variables (depicted by green rectangles) and tooth loss as the outcome (depicted by the blue rectangle). Confounding variables, indicated by gray rectangles, influence the exposure and outcome, while pink ovals represent mediating factors. Unobserved variables are denoted by an oval with a dotted line. By closing all biasing paths, only the green lines remain in the diagram. The minimum adjustment set includes age, gender, education, income, alcohol consumption, smoking, depression, diabetes, stroke, and cognitive impairment.

**Supplementary Table S1**

*Results of Complete Case Analysis (Association Between Social Isolation/Loneliness and Number of Teeth)*

| **Variable** | **PRs** | **95% CI** |
| --- | --- | --- |
| Not socially isolated | 1.0 | reference |
| Socially isolated | 0.95 | 0.91–1.00 |
| Not lonely | 1.0 | reference |
| **Lonely** | **1.07** | **1.01–1.13** |

*Note.* PR = prevalence ratio, CI = confidence interval. PRs are estimated via a modified Poisson regression model with adjustments for age, gender, smoking, alcohol consumption, education, and house income.

**Supplementary Table S2**

*Association Between Social Isolation, Loneliness, and Infrequent Toothbrushing*

| **Variable** | **Model 1 ^a^** | | **Model 2 ^b^** | |
| --- | --- | --- | --- | --- |
|  | **PRs** | **95% CI** | **PRs** | **95% CI** |
| Not socially isolated | 1.0 | reference | 1.0 | reference |
| Socially isolated | 1.17 | 0.98 – 1.39 | 1.13 | 0.95 – 1.35 |
| Not lonely | 1.0 | reference | 1.0 | reference |
| Lonely | **1.59** | **1.30 – 1.93** | **1.50** | **1.23 – 1.83** |

Note. PR = prevalence ratio, CI = confidence interval.
^a^ Model 1: PRs are estimated via a modified Poisson regression model with adjustment of age, gender, smoking, alcohol consumption, education, and house income.

^b^ Model 2: Model 1 + history of depression + history of diabetes + history of stroke + cognitive impairment.

**Supplementary Table S3**

*Results of Complete Case Analysis (Association Between Social Isolation/Loneliness and Infrequent Toothbrushing)*

| **Variable** | **PRs** | **95% CI** |
| --- | --- | --- |
| Not socially isolated | 1.0 | reference |
| Socially isolated | 1.08 | 0.89–1.31 |
| Not lonely | 1.0 | reference |
| Lonely | **1.59** | **1.27–2.00** |

*Note.* PR = prevalence ratio, CI = confidence interval. PRs are estimated via a modified Poisson regression model with adjustments for age, gender, smoking, alcohol consumption, education, and house income.

**Supplementary Table S4**

*Association Between Social Isolation, Loneliness, and Difficulty in Chewing*

| **Variable** | **Model 1^a^** | | **Model 2 ^b^** | |
| --- | --- | --- | --- | --- |
|  | **PRs** | **95% CI** | **PRs** | **95% CI** |
| Not socially isolated | 1.0 | reference | 1.0 | reference |
| Socially isolated | **1.65** | **1.12 – 2.43** | **1.63** | **1.10 – 2.41** |
| Not lonely | 1.0 | reference | 1.0 | reference |
| Lonely | **3.01** | **2.02 – 4.51** | **2.94** | **1.96 – 4.43** |

Note. PR = prevalence ratio, CI = confidence interval.
^a^ Model 1: PRs are estimated via a modified Poisson regression model with adjustment of age, gender, smoking, alcohol consumption, education, and house income.

^b^ Model 2: Model 1 + history of depression + history of diabetes + history of stroke + cognitive impairment.

**Supplementary Table S5**

*Results of Complete Case Analysis (Association Between Social Isolation/Loneliness and Difficulty in Chewing)*

| **Variable** | **PRs** | **95% CI** |
| --- | --- | --- |
| Not socially isolated | 1.0 | reference |
| Socially isolated | 1.31 | 0.81–2.12 |
| Not lonely | 1.0 | reference |
| **Lonely** | **2.59** | **1.55–4.32** |

*Note.* PR = prevalence ratio, CI = confidence interval. PRs are estimated via a modified Poisson regression model with adjustments for age, gender, smoking, alcohol consumption, education, and house income.
